# Secondary Stroke Prevention in Polish Adults: Results from the LIPIDOGRAM2015 Study

**DOI:** 10.3390/jcm10194472

**Published:** 2021-09-28

**Authors:** Beata Labuz-Roszak, Maciej Banach, Michal Skrzypek, Adam Windak, Tomasz Tomasik, Miroslaw Mastej, Maciej Tomaszewski, Dimitri P. Mikhailidis, Peter P. Toth, Alberico Catapano, Kausik K. Ray, George Howard, Gregory Y. H. Lip, Fadi J. Charchar, Naveed Sattar, Bryan Williams, Thomas M. MacDonald, Peter Penson, Jacek J. Jozwiak

**Affiliations:** 1 Department of Neurology, Institute of Medical Sciences, University of Opole, 45-052 Opole, Poland; 2 Polish Mothers Memorial Hospital Research Institute, 93-338 Lodz, Poland; maciejbanach77@gmail.com; 3 Department of Preventive Cardiology and Lipidology, Medical University of Lodz, 93-338 Lodz, Poland; 4 Cardiovascular Research Centre, University of Zielona Gora, 65-046 Zielona Gora, Poland; 5 Department of Biostatistics, Faculty of Health Sciences in Bytom, Medical University of Silesia in Katowice, 41-902 Bytom, Poland; Mskrzypek@sum.edu.pl; 6 Department of Family Medicine, Jagiellonian University Medical College, 31-061 Krakow, Poland; Mmwindak@cyf-kr.edu.pl (A.W.); Mmtomasi@cyf-kr.edu.pl (T.T.); 7 Mastej Medical Center, 38-200 Jasło, Poland; Miroslaw@mastej.pl; 8 Division of Cardiovascular Sciences, Faculty of Biology, Medicine and Health, University of Manchester, Manchester M13 9PT, UK; Maciej.Tomaszewski@manchester.ac.uc; 9 Department of Clinical Biochemistry, Royal Free Hospital, University College London, London NW3 2QG, UK; Mikhailidis@aol.com; 10 Cicarrone Center for the Prevention of Cardiovascular Disease, Johns Hopkins University School of Medicine, Baltimore, MD 21287, USA; Peter.Toth@cghmc.com; 11 CGH Medical Center, Sterling, IL 61081, USA; 12 Department of Pharmacological Sciences, University of Milano and Multimedica IRCCS, 20099 Milano, Italy; Alberico.Catapano@unimi.it; 13 Department of Primary Care and Public Health, Imperial Centre for Cardiovascular Disease Prevention, Imperial College, Kensington, London W6 8RP, UK; K.Ray@imperial.ac.uk; 14 Department of Biostatistics, School of Public Health of Alabama at Birmingham, Birmingham B15 2TT, UK; GhowArd@uab.edu; 15 Liverpool Centre for Cardiovascular Science, University of Liverpool and Liverpool Heart & Chest Hospital, Liverpool L14 3PE, UK; Gregory.Lip@liverpool.ac.uk; 16 Aalborg Thrombosis Research Unit, Department of Clinical Medicine, Aalborg University, 9000 Aalborg, Denmark; 17 School of Health and Life Sciences, Federation University Australia, Ballarat, VIC 3350, Australia; F.Charchar@federation.edu.au; 18 Institute of Cardiovascular and Medical Science, University of Glasgow, Glasgow G12 8TA, UK; Naveed.Sattar@glasgow.ac.uk; 19 NIHR University College London Biomedical Research Centre, University College London and University College London Hospitals NHS Foundation Trust, London NW1 2BU, UK; Bryan.Williams@ucl.ac.uk; 20 MEMO Research, University of Dundee, Ninewells Hospital and Medical School, Dundee DD1 9SY, UK; T.M.Macdonald@dundee.ac.uk; 21 School of Pharmacy and Biomolecular Sciences, Liverpool John Moores University, Liverpool L2 2QP, UK; P.Penson@lmju.ac.uk; 22 Liverpool Centre for Cardiovascular Science, Liverpool L69 7TX, UK; 23 Department of Family Medicine and Public Health, Institute of Medical Sciences, University of Opole, 45-052 Opole, Poland; Jacek.Jozwiak@uni.opole.pl

**Keywords:** secondary stroke prevention, oral anticoagulant drugs, oral antiplatelet drugs, primary health care

## Abstract

Background: The purpose of the study was to evaluate secondary stroke prevention in Poland and its association with sociodemographic factors, place of residence, and concomitant cardiovascular risk factors. Material and methods: From all patients in LIPIDOGRAM2015 Study (*n* = 13,724), 268 subjects had a history of ischaemic stroke and were included. Results: 165 subjects (61.6%) used at least one preventive medication. Oral antiplatelet and anticoagulation agents were used by 116 (43.3%) and 70 (26.1%) patients, respectively. Only 157 (58.6%) participants used lipid-lowering drugs, and 205 (76.5%) were treated with antihypertensive drugs. Coronary heart disease (CHD) and dyslipidaemia were associated with antiplatelet treatment (*p* = 0.047 and *p* = 0.012, respectively). A history of atrial fibrillation, CHD, and previous myocardial infarction correlated with anticoagulant treatment (*p* = 0.001, *p* = 0.011, and *p* < 0.0001, respectively). Age, gender, time from stroke onset, place of residence, and level of education were not associated with antiplatelet or anticoagulant treatment. Only 31.7% of patients were engaged in regular physical activity, 62% used appropriate diet, and 13.6% were current smokers. Conclusions: In Poland drugs and lifestyle modification for secondary stroke prevention are not commonly adhered to. Educational programmes for physicians and patients should be developed to improve application of effective secondary prevention of stroke.

## 1. Introduction

Stroke is the second most common cause of death and the leading cause of adult disability in developed countries. According to data from the World Health Organization (WHO), about 17 million people worldwide suffer a stroke every year and about 5.5 million die. In Poland, the incidence of stroke is about 90,000 per year, of which 85% are ischaemic [1,2,3,4]. A history of a previous stroke is an important risk factor for subsequent cerebrovascular events. The risk of recurrent stroke is 10–12% in the first year and 5–8% in each subsequent one [5]. The frequency of recurrence depends on the subtype of stroke and is highest in patients with embolic stroke of cardiac origin and in cases with significant atherosclerotic narrowing of the carotid arteries [5,6]. According to current guidelines, in all patients with atrial fibrillation (AF) and previous stroke (i.e., CHA_2_DS_2_VASc score ≥ 2 points), oral anticoagulants should be considered. Patients with ischemic stroke who do not have AF should be treated with antiplatelets [7,8]. Secondary stroke prevention includes also lifestyle modification (regular physical activity, proper diet, body mass reduction, smoking cessation, and reducing alcohol intake), and elimination or reduction of risk factors responsible for the first stroke (in particular guideline-specified treatment of hypertension, dyslipidaemia and diabetes) [9,10,11,12,13].

Little is known about the application of secondary stroke prevention among Polish patients, with the vast majority of former studies concerning the elderly [14,15,16]. Therefore, the purpose of this study was to evaluate the use of oral antiplatelet (OAP) and anticoagulant (OAC) drugs as secondary stroke prevention among people in Poland by using primary care patients from the LIPIDOGRAM 2015 study. We also evaluated the association of secondary stroke prevention with sociodemographic factors, place of residence, and concomitant cardiovascular (CV) risk factors. In addition, we assessed secondary stroke prevention in terms of lifestyle modification and elimination of other CV risk factors.

## 2. Materials and Methods

The LIPIDOGRAM2015 study—a nationwide observational, cross-sectional study of cardiovascular health in primary care in Poland—was design to estimate the prevalence of risk factors of atherosclerotic cardiovascular disease, frequency of cardiovascular and related disorders, and their treatment in the primary care setting of Poland. The acronym LIPIDOGRAM was constructed of two words: LIPID (lipid analysis—the main objective of the study) and GRAM (the word comes from Greek and means “what is written”). A report of the study was prepared in accordance with the STROBE Guidelines and a checklist is presented as a Supplementary Materials.

From all patients included in the LIPIDOGRAM 2015 cohort study (*n* = 13,724), 268 subjects had a history of ischaemic stroke (documented discharge from the stroke unit) and they were chosen for this analysis. Patients with history of transient ischaemic attack (TIA) were not included. Study design and rationale of The LIPIDOGRAM2015 and LIPIDOGEN2015 study—a nationwide study of cardiovascular health in primary care in Poland were described previously [17]. In brief, the study included 438 randomly selected primary care physicians in 398 practices from 16 voivodeships (major administrative regions in Poland). Each of them recruited at least 30 consecutive individuals >18 years old, who were under the care of a physician–investigator and sought care for any medical reason in the 4th quarter of 2015, or 1st and 2nd quarter of 2016. All patients were of Caucasian Polish ethnicity. The main exclusion criteria were dementia and/or psychiatric disease. To minimize potential sources of bias, each investigator–physician received individual training related to the testing procedure and methodology. During the data collection period, consultations with the main investor were also possible.

For each patient recruited for the study we completed a validated questionnaire including demographic data (age, sex, place of residence, and level of education), history and treatment of chronic cardiovascular diseases (CVD) (ischaemic and haemorrhagic stroke, hypertension, coronary heart disease (CHD), myocardial infarction (MI), AF, diabetes mellitus (DM), dyslipidaemia, familial hypercholesterolaemia, chronic kidney disease (CKD)), data on lifestyle (diet, physical activity, smoking status, alcohol intake), and CVD family history [17,18,19]. Place of residence (i.e., habitual residence at the time of examination) was classified as rural or urban. People were considered as rural residents when they were living in a settlement unit with concentrated or dispersed buildings along with existing agricultural functions or related service or tourist functions, without municipal rights or the status of a city. Whereas people were regarded as urban residents when they were living in a settlement unit with a predominance of compact development and non-agricultural functions, having municipal rights or the status of a city granted in the manner specified by the relevant act (Act on official names of places and physiographic objects, 29 August 2003, Poland). The physicians completed questionnaires based on the patient interview and data from medical records. The proper, antiatherogenic diet was defined as a Mediterranean-like diet rich in fruits, vegetables, including legumes, fibre, polyunsaturated fatty acids, nuts, and fish, with exclusion or limitation of simple sugars, red meat, dairy products, and saturated fatty acids. Regular physical activity was defined as 30–60 min of moderate activity for ≥5 days per week. Alcohol consumption was classified as none, moderate (up to ≤100 g/week or ≤15 g/day), or high (>100 g/week or >15 g/day), and smoking status as never, current, or in the past. To avoid missing data, only essential information was collected. In this way, efforts were made to minimize the burden on doctors and patients. There was also an effort to focus on routinely collected data.

For all recruited patients, anthropometric measurements were recorded (height, body weight, waist circumference, and hip circumference). BMI (body mass index) was calculated based on the height measurements in metres and body mass measurements in kilograms (kg/m^2^). The WHR (waist hip ratio) was calculated as the ratio of the waist circumference to the hip circumference. From all enrolled patients, serum samples were obtained after at least 12 h of fasting to obtain fasting glucose, glycosylated haemoglobin (HbA1C), and lipid profile. On the same day, measurements of blood pressure (BP) and heart rate (HR) were performed.

The collected blood samples were transferred in cooled containers to a central laboratory (Silesian Analytical Laboratories in Katowice, Poland). Measurements of total cholesterol (TC), triglycerides (TG), high-density lipoprotein cholesterol (HDL-C), and low-density lipoprotein cholesterol (LDL-C) were performed and carried out using Siemens Advia 1800 analyser and Siemens reagents (Munich, Germany). Fasting glycaemia was measured using Bionime glucometers (Taichung City, Taiwan) and Rightest strip tests (Taichung City, Taiwan). HbA1c was assessed using high-performance liquid chromatography (HPLC) performed by Variant II Turbo (Bio-Rad, Hercules, CA, USA).

All individuals signed an informed consent form to participate in the study and gave their permission to use anonymous questionnaire data and the results of their laboratory tests. The LIPIDOGRAM2015 Study was approved by the Bioethical Commission of the Chamber of Physicians on 2 December 2015 (No. K.B.Cz.-0018/2015).

### Statistical Analyses

Analyses were performed using SAS software, version 9.4 (SAS Institute Inc., Cary, NC, USA). The quantitative data were presented using the mean and the standard deviation or median and interquartile range for normally and non-normally distributed variables, respectively. The normality assumption was tested with the use of Shapiro–Wilk test. The quantitative variables were compared with the Student’s *t*-test and Wilcoxon–Mann–Whitney test as appropriate. Qualitative data were expressed as frequencies and percentages, and the significance of between group differences was calculated by means of the χ2 test. To assess the trend of stroke prevention according to the level of education the Cochrane-Armitage test for trend was used. Odds ratios (OR’s) with 95% confidence intervals (95% CI’s) were calculated by univariable logistic regression to assess the association of different factors with applied agents.

## 3. Results

Among all the LIPIDOGRAM2015 Study participants (*n* = 13,724), 268 (2%) subjects, 141 women and 127 men, had a history of an ischaemic stroke and they were included in this analysis. Characteristics of the study group are presented in Table 1. The mean age of participants was 67.7 ± 9.4 years, similar for both men and women (66.6 ± 8.3 vs. 68.7 ± 10.2 years *p* = 0.061, respectively). The frequencies of concomitant cardiovascular risk factors in the study group are summarized in Table 2.

Among the study group, only 165 subjects (61.6%) used at least one drug as part of a secondary prevention regimen. Antiplatelet agents were regularly used by 116 patients (43.3%), and oral anticoagulants by 70 subjects (26.1%). Antiplatelet and anticoagulant therapy was simultaneously used by 21 patients (7.8%) (Table 3). A total of 103 subjects (38.4%) were not being treated with antiplatelet agents or anticoagulants. Among participants with AF (*n* = 55; 20.5%), 35 (63.6%) used anticoagulants: 19 participants (54.3%) were receiving vitamin K antagonists (VKAs) and 16 participants (45.7%) received non-vitamin K antagonist oral anticoagulants (NOACs).

We also evaluated frequency of cardiovascular risk factor modification: 157 subjects (58.6%) used hypolipidaemic drugs (statin and/or fibrate and/or ezetimibe), 205 (76.5%) were treated with antihypertensive drugs, and 82 (30.6%) received antiglycaemic medications (Table 3). The median and interquartile range of systolic (SBP) and diastolic BPs (DBP) in patients on antihypertensive therapy (*n* = 205) were 140 mmHg (130– 150) and 80 mmHg (75–90), respectively, and were not significantly different from SBP (*p* = 0.910) and DBP (*p* = 0.797) in patients without antihypertensive therapy *(n* = 7): 140 mmHg (120–150) and 80 mmHg (70–89), respectively.

Patients taking lipid-lowering drugs had significant lower values of TC and LDL than subjects without such therapy (TC: 177.7 mg/dl (149.7–202.2) vs. 212.4 mg/dl (192.6–241.1), *p* < 0.001; LDL: 104 mg/dl (79–132) vs. 154 mg/dl (129–176), *p* < 0.001), whereas there were no statistically significant differences in HDL and TG (HDL: 49.0 mg/dl (38.6–60.0) vs. 45.8 mg/dl (42.3–57.5), *p* = 0.896; TG: 121.4 mg/dl (97.6–187.9) vs. 127.0 mg/dl (95.2–187.5), *p* = 0.669). Patients with DM using antiglycaemic medications had similar fasting glucose to those without such treatment (122 mg/dl (110–158) vs. 125 mg/dl (118–134), *p* = 0.892). Fasting glucose in patients without diagnosed DM was 100 mg/dl (91–109). HbA1C in patients with DM was 7.1% (6.2–7.7) and in patients without DM it was 5.7% (5.4–5.9), *p* < 0.001 (the data were presented as median and interquartile range).

The percentage of women after stroke using antiplatelet agents and/or anticoagulants (58.9%; *n* = 83) was similar as the corresponding percentage of men after stroke (64.6%; n = 82) (*p* = 0.338). The age of the respondents did not affect the prevalence of secondary stroke prevention; mean age of participants using antiplatelet agents and/or anticoagulants was similar to the age of participants without pharmacological stroke prevention (68.2 ± 9.6 and 66.8 ± 8.9 years, respectively, *p* = 0.206). Most of the study group lived in urban areas (54.1%; n = 145). There were no differences in using antiplatelet agents and/or anticoagulants between urban (62.8%; *n* = 91) and rural (60.2%; *n* = 74) residents (*p* = 0.663). No correlation was found between the frequency of use of antiplatelet agents and/or anticoagulants and the level of education (the Cochran–Armitage test for trend: *p* = 0.573).

Among all of the cardiovascular risk factors, CHD and dyslipidaemia were significantly associated with antiplatelet drug treatment (*p* = 0.047 and *p* = 0.012, respectively), and AF history, CHD, and previous MI with anticoagulant treatment (*p* = 0.001, *p* = 0.011, and *p* < 0.0001, respectively), see Table 4. The associations of different factors with antihypertensive, hypolipidaemic, and antiglycaemic treatment are summarized in Table 5.

## 4. Discussion

Based on this analysis of the LIPIDOGRAM2015 Study, we demonstrate that approximately 60% of examined patients with a history of a previous stroke applied anticoagulants or antiplatelets as secondary pharmacological prevention. Moreover, the patients used vitamin K antagonists more often than NOAC, and acetylsalicylic acid (ASA) was the most commonly used antiplatelet drug.

This percentage is similar to that which had been described in our previous study concerning respondents (aged 65 years and older) from the PolSenior project, carried out between April 2008 and April 2012 [15]. However, the types of medication used in secondary stroke prevention has changed. Today, many more patients are taking oral anticoagulants (26.1% vs. 5.8% previously), including NOACs, which accounts for nearly half of all OACs prescribed. Almost 65% of included patients after stroke with AF history used anticoagulants, and this percentage was much higher than in our previous study (20%) [14,15]. This still demonstrates a large gap in care and a critical need for widespread educational programs both for physicians and patients in order to improve the secondary prevention of ischemic stroke in Poland.

Among antiplatelet agents, ASA is the most frequently used drug in Poland, similarly to other countries [14]. The second most frequently used antiplatelet drug was clopidogrel, and here the situation has changed (previously ticlopidine was used more often) [14,15]. Dipyridamole was not at all used by our study group, similarly to that observed almost ten years ago. This drug is recommended in combination with ASA as a very effective agent for secondary stroke prevention [20,21,22,23]. Unfortunately, it is not dispensed in Polish pharmacies. Dipyridamole is widely used in other European countries. For instance, in Sweden it is prescribed in more than 10% of people discharged from the hospital after an ischaemic stroke [20].

Among VKAs, acenocoumarol is still the most widely used drug in Poland, which is in contrast to the United States and Western Europe, where warfarin is much more popular [14,15]. Similar to our previous paper, we found no association between use of antiplatelet agents and/or anticoagulants and age, sex, place of residence, or level of education [15].

Secondary stroke prevention includes not only pharmacological treatment but also modification of lifestyle and other cardiovascular risk factors. Hypertension is a major risk factor for ischemic stroke, and its treatment can significantly reduce the risk of recurrent ischemic stroke [23,24]. The current guidelines recommend starting antihypertensive therapy in patients with SBP > 140 mmHg and/or DBP > 90 mmHg [23]. Thiazide diuretics alone or in combination with an angiotensin-converting enzyme inhibitor (ACEI) should be used in patients after stroke as a first-line treatment [23,24,25]. In our study, antihypertensive drugs were taken by 76.5% of respondents; there were mostly diuretics (37.7%) and ACE inhibitors (36.9%). However, this therapy was not always effective; the median SBP and DBP in patients on antihypertensive therapy was 140 mmHg and 80 mmHg, respectively.

DM is a major risk factor for ischemic stroke and an increased HbA1c level is associated with increased stroke risk in people with and without DM [26]. In the study group, the control of diabetes was suboptimal. The average fasting glucose concentration in patients with diabetes was about 120 mg/dl and HbA1C was 7.1%, which are higher than the recommended levels in recent guidelines [23].

High dose, high intensity statin therapy should be considered in all patients after an ischaemic stroke (based on very high cardiovascular risk). Adjuvant therapy with ezetimibe or fenofibrate may also be indicated [27,28,29,30]. Statins were taken by only approximately 60% of participants, but the average LDL concentration was still elevated (mean 104 mg/dl), although not as high as in people not treated with lipid lowering drugs (154 mg/dl). It is worth noting that in this study group of patients, the frequency of heterozygous familial hypercholesterolaemia was much more frequent (3%) than in the general population (0.2–0.5%) (Table 4).

Although there are no clear recommendations concerning weight reduction in obese patients after stroke, obesity is associated with other stroke risk factors such as hypertension, diabetes, and dyslipidaemia [21,22]. The average BMI in the study group was nearly 30 kg/m^2^ and more than 80% of respondents were overweight or obese. Regular physical activity is a known factor modifying other CV risk factors; it reduces blood pressure, lowers serum levels of atherogenic lipoproteins and glucose, helps in maintaining proper body weight; it also reduces the risk of recurrent stroke itself [25]. In this study, only one third of stroke patients were engaged in regular physical activity, as they admitted in the questionnaire. Smoking increases the risk of cerebrovascular incident 2–4 fold, so stroke survivors should be advised to stop smoking [9]. The percentage of current smokers was 13.6% in our study. In addition, 1.5% of respondents admitted that they consume high doses of alcohol. Another important lifestyle factor is an appropriate low-fat diet. Some clinical trials revealed the benefits of use of the Mediterranean diet or DASH (Dietary Approach to Stop Hypertension) diet in reducing cardiovascular risk, including stroke risk [31,32,33]. In our study, 62% of the respondents tried to use an appropriate diet.

### Limitations

It was surprising for us that about 40% of people after stroke did not admit antithrombotic and/or hypolipidemic drugs. Secondary prevention—both pharmacological and lifestyle modification—should be ordered at discharge from stroke unit and then continued ambulatorily by neurologists and/or primary care physicians. The reasons for discontinuing therapy could not be established from available data. One of reasons could be long time from the stroke (6.6 ± 5.6 years) and patients’ non-compliance.

The study group was relatively small but representative for the primary health care and the whole country (patients were recruited from every voivodeship). However, the average age of the respondents was about 5 years lower than the average age of patients hospitalized in Poland due to a first stroke [34]. The study did not include the most disabled patients who stayed at home and were not able to consult a general practitioner. Evaluation of smoking habits and alcohol consumption by questionnaire has limited validity. We analysed the association of different factors with used medications, including antiplatelets, anticoagulants, hypolipidaemic drugs, antidiabetics, and antihypertensive drugs, but it should be emphasized that this relationship cannot show a causal relationship due to the characteristic of cross-sectional study.

## 5. Conclusions

Poland therapeutic drugs and lifestyle modification for secondary stroke prevention are not commonly adhered to. Educational programmes both for physicians and patients should be developed to improve application of effective secondary prevention of stroke. Procedures of patients’ adherence monitoring should be implemented.

## Figures and Tables

**Table 1:** Characteristics of the study group (*n* = 268). Data presented as the number (*n*) and percentage (%) value or mean and standard deviation (SD).

Characteristics		Patients with IS *n* = 268
Gender *n* (%)	Female	141(52.6%)
	Male	127 (47.4%)
Education *n* (%)	Primary	59 (22%)
	Vocational	95 (35.5%)
	Secondary	88 (32.8%)
	Higher	26 (9.7%)
Place of residence *n* (%)	Urban	145 (54.1%)
	Rural	123 (45.8%)
Data presented as mean ± SD		
Age (mean ± SD), years		68 ± 9
BMI, kg/m^2^		29.5 ± 4.9
WHR		0.98 ± 0.1
Duration from acute stroke, years		6.6 ± 5.6
SBP, mmHg		138.2 ± 18.7
DBP, mmHg		81 ± 10.6
HbA1C, %		6.2 ± 1.14
TC, mg/dl		191.6 ± 53.8
HDL-C, mg/dl		51 ± 15.5
TG, mg/dl		151 ± 91.6
LDL-C, mg/dl		119.6 ± 47.4
Fasting glucose, mg/dl		115 ± 37.55
HR, bpm		74 ± 10

IS—ischaemic stroke; BMI—body mass index; WHR—waist hip ratio; SBP—systolic blood pressure; DBP—diastolic blood pressure; HbA1C—glycosylated haemoglobin; TC—total cholesterol; HDL-C—high-density lipoprotein cholesterol; LDL-C—low-density lipoprotein cholesterol; TG—triglycerides; HR—heart rate; bpm—beats per minute; SD—standard deviation.

**Table 2:** Lifestyle and cardiovascular risk factors in the study group (*n* = 268). Data presented as the number (*n*) and percentage (%) value.

Risk Factor		*n* (%)
History of HS		5 (1.9%)
CHD		111 (41.4%)
Previous myocardial infarction		49 (18.3%)
History of AF		55 (20.5%)
Dyslipidaemia		186 (69.4%)
Familial hypercholesterolaemia		8 (3%)
DM		87 (32.5%)
HT		212 (79.1%)
CKD		13 (4.9%)
Overweight and/or obesity (BMI > 25 kg/m^2^)		222 (82.8%)
Smoking	current	36 (13.4%)
	in the past	97 (36.2%)
	never	135 (50.4%)
Alcohol consumption	never	122 (45.5%)
	moderate	142 (53%)
	high	4 (1.5%)
Regular physical activity		85 (31.7%)
Use of antiatherogenic diet		166 (62%)

HS—haemorrhagic stroke; CHD—coronary heart disease; AF—atrial fibrillation; DM—diabetes mellitus; HT—hypertension; CKD—chronic kidney disease; BMI—body mass index.

**Table 3:** Frequency of use of particular medications in the study group (*n* = 268).

Medications	International Drug Name	Number of Respondents Using the Drug *n* (%)
**Antiplatelets** *n* = 116 (43.3%)	ASA	95 (81.9%)
	Clopidogrel	18 (15.5%)
	Ticlopidine	12 (10.3%)
**Anticoagulants** *n* = 70 (26.1%)	Warfarin	22 (28.6%)
	Acenocumarol	20 (31.4%)
	NOAC	28 (40%)
**Hypolipidaemic drugs**		157 (58.6%)
Statins		137 (58.6%)
Fibrates		12 (4.5%)
Ezetimib		4 (1.5%)
FDCs for dyslipidaemia		4 (1.5%)
**Antidiabetics**		82 (30.6%)
**Antihypertensive drugs**		205 (76.5%)
ACE inhibitors		99 (36.9%)
BBs		82 (30.6%)
CCBs		46 (17.2%)
Diuretics		101 (37.7%)
ARBs		42 (15.7%)
FDCs for hypertension		39 (14.6%)
Other antihypertensive drugs		31 (11.6%)

ASA—acetylsalicylic acid; NOAC—non-vitamin K antagonist oral anticoagulants; ACE—angiotensin converting enzyme; BBs—beta-blockers; CCBs—calcium channel blockers; ARBs—angiotensin II receptor blockers; FDCs—fixed dose combination pills.

**Table 4:** Association of different factors with antiplatelets and/or anticoagulant treatment (*n* = 268). Data are presented as OR (95%CI) and *p*-value.

Factor	Antiplatelets or Anticoagulants		Antiplatelets		Anticoagulants	
	OR (95% CI)	*p*	OR (95% CI)	*p*	OR (95% CI)	*p*
HS	0.93 (0.15–5.69)	0.942	1.99 (0.33–12.11)	0.455	<0.001 (<0.001–>99.99)	0.958
CKD	1.43 (0.43–4.76)	0.562	1.56 (0.51–4.78)	0.434	1.83 (0.58–5.78)	0.305
CHD	2.35 (1.39–3.96)	0.001	1.65 (1.01–2.69)	0.047	2.59 (1.48–4.52)	0.001
Previous myocardial infarction	1.93 (0.97–3.85)	0.061	1.20 (0.64–2.23)	0.568	2.33 (1.22–4.47)	0.011
AF	4.73 (2.13–10.49)	<0.0001	1.34 (0.74–2.44)	0.330	8.90 (4.61–17.19)	<0.0001
Dyslipidaemia	1.61 (0.95–2.73)	0.078	2.02 (1.17–3.49)	0.012	0.79 (0.44–1.42)	0.436
Familial hypercholesterolaemia	1.04 (0.24–4.45)	0.956	0.78 (0.18–3.34)	0.738	1.73 (0.40–7.43)	0.462
DM	1.61 (0.94–2.76)	0.086	1.66 (0.99–2.78)	0.054	1.33 (0.75–2.35)	0.331
HT	1.26 (0.69–2.29)	0.445	1.49 (0.81–2.74)	0.200	0.62 (0.33–1.17)	0.137
Overweight/obesity BMI > 25	0.83 (0.43–1.61)	0.576	0.80 (0.42–1.52)	0.495	1.15 (0.55–2.41)	0.708
Current smoking	0.98 (0.48–2.01)	0.952	0.93 (0.46–1.89)	0.833	0.93 (0.416–2.09)	0.869
Older age > 70 years	1.47 (0.87–2.49)	0.149	1.06 (0.64–1.76)	0.820	1.42 (0.81–2.48)	0.225
Male gender	1.27 (0.78–2.09)	0.338	1.54 (0.95–2.50)	0.083	0.84 (0.49–1.46)	0.546
Secondary/higher education	1.37 (0.83–2.26)	0.222	0.98 (0.60–1.60)	0.932	1.39 (0.81–2.41)	0.236
Urban residence	1.12 (0.68–1.83)	0.663	1.02 (0.63–1.65)	0.953	0.93 (0.54–1.61)	0.807
Regular physical activity	1.31 (0.77–2.24)	0.323	1.25 (0.75–2.10)	0.396	0.82 (0.45–1.49)	0.511
Use of antiatherogenic diet	0.81 (0.48–1.34)	0.408	0.89 (0.54–1.46)	0.638	0.76 (0.44–1.33)	0.337
Duration from stroke >1 year	0.79 (0.42–1.48)	0.456	1.21 (0.65–2.23)	0.548	0.69 (0.36–1.33)	0.272

HS—haemorrhagic stroke; CHD—coronary heart disease; AF—atrial fibrillation; DM—diabetes mellitus; HT—hypertension; CKD—chronic kidney disease; BMI—body mass index; OR-odds ratio; CI-confidence interval

**Table 5:** Association of different factors with antihypertensive, hypolipidaemic and antidiabetic treatment (*n* = 268). Data are presented as OR (95%CI) and *p*-value.

Risk Factor	Antihypertensive Drugs		Hypolipidaemic Drugs		Antidiabetics	
	OR (95% CI)	*p*	OR (95% CI)	*p*	OR (95% CI)	*p*
HS	0.45 (0.07–2.77)	0.392	1.06 (0.17–6.46)	0.948	3.49 (0.57–21.32)	0.175
CKD	1.73 (0.37–8.02)	0.484	1.63 (0.49–5.42)	0.428	1.01 (0.30–3.37)	0.989
CHD	2.82 (1.48–5.35)	**0.002**	1.56 (0.95–2.58)	0.080	1.78(1.05–3.01)	**0.031**
Previous myocardial infarction	2.06 (0.88–4.85)	0.098	1.14 (0.61–2.16)	0.678	2.67 (1.41–5.03)	**0.003**
AF	1.74 (0.80–3.78)	0.165	0.81 (0.45–1.48)	0.496	1.54 (0.83–2.86)	0.173
Dyslipidaemia	2.68 (1.49–4.81)	**0.001**	-	-	1.42 (0.79–2.54)	0.241
Familial hypercholesterolaemia	2.192 (0.265–18.161)	0.467	-	-	1.38 (0.32–5.89)	0.668
DM	3.19 (1.53–6.63)	0.002	1.54 (0.91–2.62)	0.111	-	-
HT	-	-	2.98 (1.62–5.49)	**<0.001**	5.82 (2.23–15.20)	**<0.001**
Overweight/obesity BMI > 25	3.60 (1.84–7.04)	**<0.001**	1.52 (0.81–2.88)	0.196	4.35 (1.65–11.47)	**0.003**
Current smoking	1.318 (0.548–3.173)	0.538	1.129 (0.550–2.317)	0.741	0.61 (0.27–1.40)	0.245
Age > 70 years	1.13 (0.62–2.05)	0.688	0.641 (0.386–1.064)	0.085	1.56 (0.92–2.67)	0.101
Male gender	1.17 (0.66–2.06)	0.593	0.762 (0.468–1.241)	0.275	1.16 (0.69–1.96)	0.569
Secondary/higher education	0.76 (0.43–1.35)	0.352	1.150 (0.703–1.883)	0.578	0.70 (0.41–1.20)	0.192
Urban residence	1.01 (0.57–1.775)	0.980	1.003 (0.616–1.634)	0.989	1.12 (0.67–1.89)	0.664
Regular physical activity	0.99 (0.54–1.83)	0.995	0.713 (0.424–1.199)	0.202	0.92 (0.53–1.62)	0.774
Use of antiatherogenic diet	0.77 (0.42–1.39)	0.378	0.808 (0.488–1.337)	0.407	0.45 (0.27–0.77)	**0.004**
Duration from stroke >1 year	1.37 (0.70–2.71)	0.359	1.106 (0.60–2.03)	0.744	1.15 (0.59–2.23)	0.686

HS—haemorrhagic stroke; CHD—coronary heart disease; AF—atrial fibrillation; DM—diabetes mellitus; HT—hypertension; CKD—chronic kidney disease; BMI—body mass index; OR-odds ratio; CI-confidence interval

## Data Availability

The data presented in this study are available on request from the corresponding authors. The data are not publicly available due to privacy.

## Supplementary Materials

The following are available online at https://www.mdpi.com/article/10.3390/jcm10194472/s1, STROBE Statement—Checklist of items that should be included in reports of cross-sectional studies.
